# Flucast: A Real-Time Tool to Predict Severity of an Influenza Season

**DOI:** 10.2196/11780

**Published:** 2019-07-23

**Authors:** Aye Moa, David Muscatello, Abrar Chughtai, Xin Chen, C Raina MacIntyre

**Affiliations:** 1 Biosecurity Program The Kirby Institute University of New South Wales Sydney Australia; 2 School of Public Health and Community Medicine Faculty of Medicine University of New South Wales Sydney Australia; 3 College of Health Solutions and College of Public Service & Community Solutions Arizona State University Tempe, AZ United States

**Keywords:** prediction tool, influenza, risk assessment

## Abstract

**Background:**

Influenza causes serious illness requiring annual health system surge capacity, yet annual seasonal variation makes it difficult to forecast and plan for the severity of an upcoming season. Research shows that hospital and health system stakeholders indicate a preference for forecasting tools that are easy to use and understand to assist with surge capacity planning for influenza.

**Objective:**

This study aimed to develop a simple risk prediction tool, Flucast, to predict the severity of an emerging influenza season.

**Methods:**

Study data were obtained from the National Notifiable Diseases Surveillance System and Australian Influenza Surveillance Reports from the Department of Health, Australia. We tested Flucast using retrospective seasonal data for 11 Australian influenza seasons. We compared five different models using parameters known early in the season that may be associated with the severity of the season. To calibrate the tool, the resulting estimates of seasonal severity were validated against independent reports of influenza-attributable morbidity and mortality. The model with the highest predictive accuracy against retrospective seasonal activity was chosen as a best-fit model to develop the Flucast tool. The tool was prospectively tested against the 2018 and the emerging 2019 influenza season.

**Results:**

The Flucast tool predicted the severity of all retrospectively studied years correctly for influenza seasonal activity in Australia. With the use of real-time data, the tool provided a reasonable early prediction of a low to moderate season for the 2018 and severe seasonal activity for the upcoming 2019 season. The tool meets stakeholder preferences for simplicity and ease of use to assist with surge capacity planning.

**Conclusions:**

The Flucast tool may be useful to inform future health system influenza preparedness planning, surge capacity, and intervention programs in real time, and can be adapted for different settings and geographic locations.

## Introduction

Influenza is an epidemic infection that affects millions of people around the world with varying severity. It infects approximately 10% to 15% of adults and 20% to 30% of children annually [1]. In aged care facilities and within the community, estimated attack rates can be 25% or higher [2,3].

Traditionally, influenza activity is monitored through a range of national and global surveillance networks in each country and globally. The data sources include laboratories, hospitals and sentinel general practices, morbidity and mortality data from health departments, and online self-reported community surveillance such as Flu Tracking [4-7]. These data, however, are typically retrospective and have inherent time lags. They are not generally used to forecast the severity of an emerging season and may not provide early warning for facilitating preparedness and surge capacity planning. Increased hospital and health system demand during the influenza season [8-10] is a high priority for health managers because influenza epidemics result in a surge of emergency department and hospital admissions [11-13].

Various predictive tools and methods for forecasting influenza epidemics and timing of seasonal peaks for influenza have been developed [14-16]. The Centers for Disease Control and Prevention (CDC) and other institutions in the United States have developed influenza assessment tools that are made available for local-level seasonal prediction [17]. In 2016, the CDC launched FluSight on the Epidemic Prediction Initiative website to forecast weekly influenza activity [18]. Research teams submit weekly flu forecasts to FluSight, which then provides information on influenza onset week, peak week, and peak intensity as well as influenza-like illness activity during the season [19].

Using the CDC FluAid 2.0 modeling tool [17], one Australian study reported a favorable forecasting value for decision making and planning of health care services during the 2009 influenza pandemic [20]. The study found that the model predictions were comparable to actual reports from hospitals regarding hospital and intensive care admissions in the study. It was evident that timely use of modeling tools could help to inform and manage resources and surge capacity requirements at hospitals during severe seasons and pandemics [20]. Although these advanced modeling tools are useful to forecast the situation in real time, they involve complex mathematical modeling approaches that are not easily understood by health system stakeholders and may not be adaptable to other settings.

From a previous study of Australian epidemic planning and preparedness, we found that stakeholders do not apply epidemic modeling tools in routine public health practice, and they have skepticism and distrust of modeling tools. They indicated a preference for simple tools, which are easy to apply and understand. In addition, the stakeholders stated that their highest priority was surge and workforce capacity planning during the influenza season [21].

To forecast the influenza epidemic in real time and assist with surge capacity planning, we aimed to develop a simple assessment tool for early prediction of seasonal influenza severity using the surveillance data in the study.

## Methods

### Overview

The Australian influenza season generally falls between May and October, with the peak occurring between July and September [22]. Laboratory-confirmed influenza infection is a notifiable disease in Australia, and cases are reported to state and territory health authorities. National data are published by the Australian Government Department of Health. During the influenza season, the Australian Influenza Surveillance Reports provide biweekly descriptive reports of influenza activity at a national level, including updates on international influenza activity [7]. In this study, a tool was developed by fitting an algorithm to 11 years of retrospective influenza data and then testing it prospectively against the 2018 and the emerging 2019 influenza season in Australia.

### Data Sources and Parameters

Data were obtained from the following sources: (1) laboratory-confirmed influenza notifications from the National Notifiable Diseases Surveillance System (NNDSS) [5] and (2) published Australian Influenza Surveillance Reports [7] in 2016 and 2017. The National Australian Influenza Surveillance Scheme, the Australian Government, Department of Health reports and provides information regarding seasonal influenza activity, circulating viruses, and influenza vaccine information for the years studied.

When developing the models, a range of variables was considered to include in the forecast model to predict the severity of seasonal activity. These included total number of notified cases in the season using a different month (such as April, May, or June) to determine early or late season starts as well as the magnitude, viral subtypes in circulation, pediatric influenza-related deaths, reported number of influenza-related hospitalizations, intensive care admissions at a single time point, and reported influenza-associated deaths in the season. However, in early testing of more than nine variations with inclusion and exclusion of the different variables mentioned previously in models with at least three to six parameters (data not shown), several of these variables and models were excluded in the forecast model because they did not contribute to or assist in predicting seasonal influenza severity. We then selected the parameters that were associated with or contributed to the severity of influenza during the season, such as timing of season, magnitude or number of notified cases, viral strain, and influenza-associated hospitalizations or deaths in the season. In the final selections, we selected five parameters and five different models that might contribute to or assist in predicting the season’s severity to test the best-fit model for the tool. The data applied were for the Australian influenza season in the study; therefore, month 1 was defined as May (the first month of the beginning of influenza season) and month 2 as June in the models. The five parameters considered are discussed subsequently.

#### Timing of Seasonal Onset

This was used to define the onset of a season (being an early or late onset to see any impact on seasonal severity) using notifications in month 1 or month 2 for a given year. For this parameter, data were retrieved from the NNDSS [5].

#### Relative Magnitude of Influenza Activity

The relative magnitude was the relative rate of influenza notifications in month 1 or month 2 compared with the past five years’ average. Data were obtained from the NNDSS [5].

#### Dominant Strain in Circulation

This was defined as the viral strain that was 50% or more of the circulating strains or the highest proportion strain circulating during the season. Severity and scoring criteria were assigned based on reported studies [10,23,24]. Data were obtained from the Australian Influenza Surveillance Report from the Department of Health [7]. A novel strain (categorized as the most severe strain) and A(H1N1)pdm09 in 2009 were treated as a novel or pandemic strain for that year, followed by influenza A(H3N2), influenza B, and influenza A(H1N1). Due to the inclusion of prepandemic years (2007 and 2008), the influenza A(H1N1) subtype was included in the study, although it has not been circulated widely since 2009.

#### Vaccine Mismatch

A documented mismatch of a vaccine strain is a change in the amino acid in the hemagglutinin or neuraminidase surface proteins of dominant strains of influenza viruses in circulation and the southern hemisphere influenza vaccine strains recommended by the World Health Organization (WHO) for a given season. Reports of a vaccine mismatch were retrieved from the Australian Influenza Surveillance Report-WHO Collaborating Centre for Reference and Research on Influenza [7]. The vaccine mismatch information is available at the earliest around month 1 (May) or month 2 (June) if there is delayed reporting during the season.

#### Early Season Deaths

Data on early season deaths (rate of notified influenza-associated deaths early in the season per 100,000 population) were obtained from the report of influenza-associated deaths notified to the NNDSS at the end of July in the current influenza season from the Australian Influenza Surveillance Report [7]. Data from July were used to account for delayed reporting of deaths in the administrative system in general. Population data were obtained from the Australian Bureau of Statistics from the Australian Government [25]. Morbidity and mortality burden could demonstrate the severity of influenza infection; thus, we applied mortality (deaths data) to predict seasonal severity in the models.

Severity prediction of influenza is complex and multifocal in nature, and more than one factor would have been attributed to the severity in the season. In our models, we assumed that each parameter contributed equally to the prediction of seasonal severity. Each model contained either four or five parameters as listed in Table 1. Model 1 was chosen as a reference model, and the other models (models 2-5) resulted from the removal or replacement of a parameter from the reference model (model 1).

The five models tested were:

1. Model 1: consisted of all five parameters (parameters 1-5 as shown in Table 1) and was used as a reference model in the study.
2. Model 2: consisted of four parameters (parameters 2-5), with removal of the seasonal onset column from the reference model.
3. Model 3: consisted of four parameters (parameters 1-4), with removal of notified influenza-associated deaths from the reference model.
4. Model 4: consisted of all five parameters (parameters 1-5) as in Model 1; however, a different scoring method was used to score the dominant strain in the model.
5. Model 5: consisted of all five parameters (parameters 1-5). In this model, for a designated month, month 2 was used instead of month 1 to calculate the ratio of notifications for both parameters 1 and 2 in the model.

Then, we considered predefined criteria to score parameters in the model. A simple, discrete linear scoring method, with 0 being the lowest and 4 being the highest score, was used to score each parameter (Table 1).

A score of 0 was regarded as no impact, and a higher score indicated a stronger prediction of severity for the season. For any given year, each parameter was given a score based on its value. The score increased with a higher risk value of the parameter. The scores for each parameter were summed to give a total score for each year in the model. The maximum possible score given in the model ranged from 16 to 20, depending on the number of parameters included in the model. For example, in model 1, the maximum possible score would be the sum of the highest score of 4 for each parameter multiplied by 5 parameters, which equals 20.

### Scoring of Models and Selection of the Best-Fit Model

In developing the Flucast tool, data available each year from the influenza surveillance reports and laboratory-confirmed influenza notifications from the NNDSS were compiled to predict and categorize annual influenza seasonal severity in the models [5,7]. The historical data from the past 11 consecutive years (2007-2017 including the pandemic in 2009) were applied. We trained the model using data from 2007 to 2017 retrospectively and tested it with 2018 data in real time as the 2018 influenza season was emerging at the time of the study.

As per the scoring criteria in Table 1, data were scored and total scores were calculated for an individual year in the five models. Then, the severity index percentage was calculated for each year. The formula for calculating the severity index for any given year in the model was:

severity index (%) = (total score obtained from the parameters / maximum score in the model) * 100

Lastly, we calibrated the severity index against seasonal severity (with reference to historical data from the surveillance reports, knowing which past seasons were mild, moderate, or severe in Australia). We considered the bottom 30% as mild, middle 30% as moderate, the next 30% as severe, and the final 10% as very severe, and severity index was categorized as a mild season (<30%), moderate (30% to 59%), severe (60% to 89%), or very severe season (≥90%). The severity index resulting from the model outputs were then applied accordingly to calibrate the seasonal severity.

**Table 1:** Parameters and scoring criteria of the influenza prediction models (Australian Influenza Surveillance Reports [7] and NNDSS [5]).

Parameter and criteria			Scores
**1. Timing of seasonal onset: Ratio of laboratory-confirmed influenza notifications in month 1 to preceding four months’ average for a given year [models 1, 3, & 4] or ratio of laboratory-confirmed influenza notifications in month 2 to preceding four months’ average for a given year [model 5], if…**			
	≤1		0
	>1 to 1.5		1
	>1.5 to 2		2
	>2 to 2.5		3
	>2.5		4
**2. Relative magnitude of influenza activity: Ratio of laboratory-confirmed influenza notifications in month 1 for a given year compared with last 5 years’ average for the same period [models 1, 2, 3, & 4] or ratio of laboratory-confirmed influenza notifications in month 2 for a given year compared with last 5 years’ average for the same period [model 5], if…**			
	≤1		0
	>1 to 1.5		1
	>1.5 to 2		2
	>2 to 2.5		3
	>2.5		4
**3. Dominant strain in circulation: Viral strain comprising ≥50% of circulating strains or the highest proportion circulating in the season**			
	**For scoring in models 1-3 and 5**		
		B or A(H1N1)	1
		A(H1N1)pdm09	2
		A(H3N2)	3
		Novel strain	4
	**For scoring in model 4**		
		A(H1N1)	1
		B	2
		A(H3N2) or A(H1N1)pdm09	3
		Novel strain	4
**4. Vaccine mismatch in the season: Documented vaccine mismatch with the dominant strain in the season**			
	No mismatch		1
	Mismatch in 1 strain only		2
	Mismatch in >1 but not all strains		3
	Mismatch in all strains		4
**5. Early season deaths: Rate of notified influenza-associated deaths per 100,000 population at the end of July in the current season**			
	≤0.01		1
	>0.01 to 0.05		2
	>0.05 to 0.1		3
	>0.1		4

From the five potential models, the model with the best fit against the known severity of the past 11 seasons was selected as the final model for the Flucast tool. Thus, the best-fit model would be the model that would give the highest accuracy of seasonal prediction among the five.

Independent data on morbidity and mortality were used to classify and validate the annual seasonal impact for the years included as very severe, severe, moderate, or mild [5,26], which provided accuracy and classification for forecast severity. However, the results from a recent Australian study were available up to 2013 [26]; thus, the estimated seasonal impact for the years 2014 to 2017 were validated using records from the National Influenza Surveillance Reports [7]. In Australia, the years 2008, 2010, 2011, and 2013 to 2016 had moderate or mild seasonal activity; 2007, 2012, and 2017 were severe seasons. In general, a pandemic can occur at any time point, and the 2009 pandemic year in Australia somehow coincided with the seasonal period, but only a few months earlier than the usual time in the country.

Using the final chosen model, we developed and implemented the online Flucast tool, which allows users to enter information obtained from the real-time surveillance data to predict the severity of the current influenza season. Input data required for parameters, the procedure for calculation, and links to the sources of Australian data are also provided on the webpage. Options to choose an answer for each parameter are provided in the drop-down lists. Once all answers are filled, a severity index percentage with estimated seasonal severity appears on the thermometer indicator as the final output of the tool. The Flucast tool online page is incorporated in a designated website, and the Web link to the online site is presented in the study.

The Flucast tool was developed and validated in 2016 and 2017 in Australia using the local data. A modified version of the Flucast tool has also been developed, which is adapted for other settings, such as countries in the southern or northern hemisphere with regular influenza seasonal patterns. For these modified versions, we assumed that the influenza season falls between May and October for the southern hemisphere countries and November and April for countries in the northern hemisphere.

## Results

### Scoring of Models and Selection of the Best-Fit Model

Using the available Australian data for past influenza seasons and prospectively for the 2018 influenza season, all five models were scored using the scoring criteria and forecasted the seasonal severity for each year. An example of the scoring method is shown in Table 2 for model 1 (the reference model). Final scores for models 2 to 5 are presented in Multimedia Appendices 1-4.

The outputs from the five proposed alternative models provided a reasonable estimation of influenza severity. All models except model 3 predicted well for the severe seasons of 2007 and 2012, as well as 2017 in Australia. All five models identified the 2009 pandemic year as a very severe season. There were some variations across the models in predicting moderate and mild seasons.

**Table 2:** Scoring method for model 1 (from Australian Influenza Surveillance Reports [7] and NNDSS [5]).

Year	Actual impact of season	Parameters^a^					Total score (max=20)	Severity index^b^, %
		Timing of seasonal onset (score)	Relative magnitude of influenza activity (score)	Dominant strain (score)	Vaccine mismatch in the season (score)	Early season deaths (score)		
2007	Severe	1.1 (1)	1.3 (1)	A/H3N2 (3)	All strains (4)	18^c^ (3)	12	60
2008	Moderate	1.7 (2)	2.8 (4)	B (1)	1 strain (2)	3^c^ (1)	10	50
2009	Very severe (pandemic)	13.7 (4)	19.3 (4)	Novel/pandemic strain or A/H1N1pdm09 (4)	All strains (4)	61 (4)	20	100
2010	Mild	1.2 (1)	0.3 (0)	A/H1N1pdm09 (2)	None (1)	2 (1)	5	25
2011	Moderate	1.1 (1)	1.4 (1)	A/H1N1pdm09 (2)	None (1)	10 (2)	7	35
2012	Severe	2.5 (3)	1.4 (1)	A/H3N2 (3)	>1 but not all strains (3)	23 (3)	13	65
2013	Moderate	1.1 (1)	0.8 (0)	A/H1N1pdm09 (2)	1 strain (2)	11 (2)	7	35
2014	Moderate	1.1 (1)	1.2 (1)	A/H1N1pdm09 (2)	1 strain (2)	22 (3)	9	45
2015	Moderate	1.5 (1)	2.9 (4)	B (1)	None (1)	46 (4)	11	55
2016	Moderate	1.0 (0)	1.5 (1)	A/H1N1pdm09 (2)	None (1)	17 (3)	7	35
2017	Severe	1.3 (1)	2.1 (3)	A/H3N2 (3)	1 strain (2)	43 (4)	13	65
2018^d^	Moderate	0.6 (0)	1.0 (0)	A/H1N1pdm09 (2)	None (1)	35 (3)	6	30

^a^Timing of seasonal onset: ratio of laboratory-confirmed influenza notifications in May/January to April average [5]; relative magnitude of influenza activity: ratio of laboratory-confirmed influenza notifications in May compared to last 5 years’ average [5]; dominant strain: dominant strain in circulation [7]; vaccine mismatch in season: vaccine mismatch with dominant strain(s) [7]; early season deaths: rate per 100,000 population of notified influenza-associated deaths at the end of July in the season [7].

^c^Severity index=total score/maximum score.

^d^Influenza-associated deaths in 2007 and 2008 were estimated by calculating the proportion (total number of notifications at the end of July/total notifications in the year) multiplied by total deaths reported by the Australian Bureau of Statistics for 2007 and 2008, accordingly.

^e^Prospective year, real-time data.

In our study, we used a simple method of scoring variables and parameters to estimate the severity of the influenza season. There were not many differences between the models; however, it indicated that the removal of notified influenza-associated deaths (model 3) gave the lowest predicted accuracy among the five models. Model 1 estimated well for all seasons retrospectively for the past 11 years and predicted the prospective 2018 season correctly when the results were validated against the actual impact of the influenza season in Australia. Thus, from the five tested models, model 1 showed the best fit for all years, accurate for 11 of 11 seasons, so it was chosen as the best-fit model for the Flucast tool. It was followed by model 2, then models 4, 5, and 3. The severity indexes and predicted seasonal activities by the models are described in Table 3. In validating the impact of actual seasons with predicted estimates, the reported data may be underestimated [27]; however, these data did show a seasonal trend for the years studied. Using the Flucast tool for real-time assessment in 2018, the tool predicted the season as moderate (severity index score of 30%) by late July. The seasonal peak occurred in late August in 2018, and a low level of seasonal activity was reported for 2018 in general [28].

Sensitivity analysis of the Flucast tool was conducted as a post hoc analysis in the study. In predicting moderate versus mild seasons using fewer parameters (less than five parameters), the results were less accurate. The sensitivity was reduced to approximately 17% in predicting moderate seasons. Also, we found that the models were 33% less sensitive in predicting a severe versus moderate season when using only four parameters (data not shown). As a result, we did not test further for mild seasons, and we concluded that the tool might not provide an accurate estimation of seasonal activity with fewer parameters. In addition, the impact of a pandemic year in the model prediction was also determined in the study. Sensitivity was tested in model 1 from 2010 to 2014 for scoring of parameter 2 in calculating the last 5 years’ average with inclusion and exclusion of 2009 to see the overall impact on seasonal prediction by the model. It was shown that seasonal predictions were almost the same, except for 2010 (data not shown).

**Figure 1:**
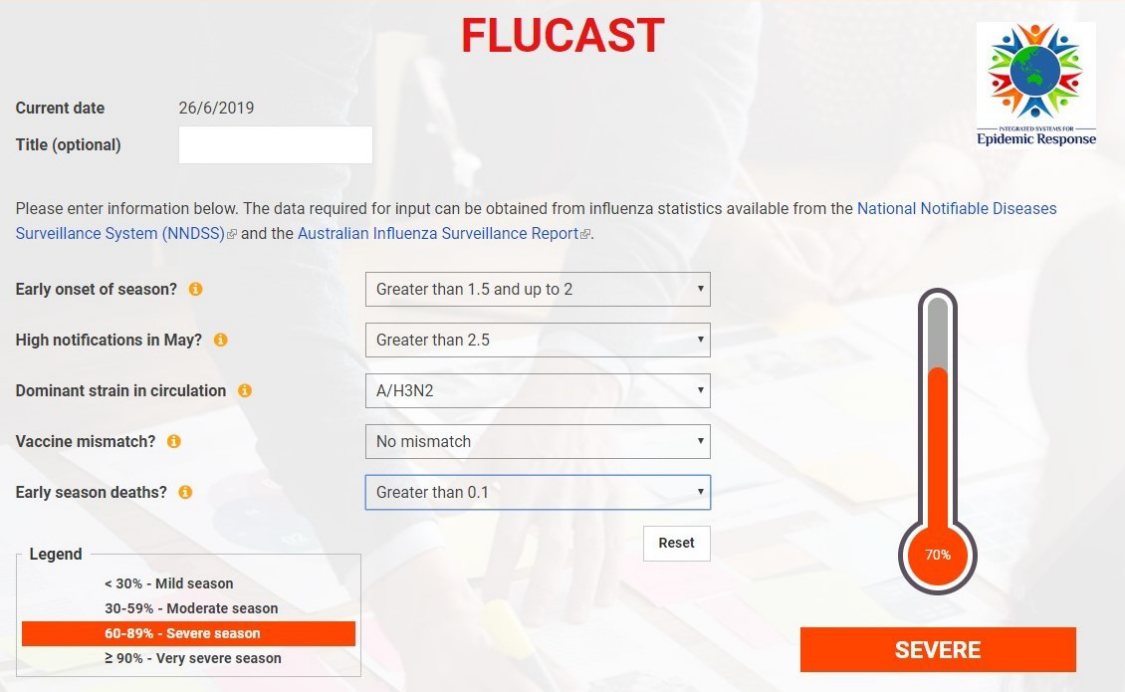
The Flucast tool (online form).

The online form of the Flucast tool and an example of the tool image (as predicted seasonal severity for 2019) is shown in Figure 1 [29]. The Flucast tool was tested using real-time data for the 2019 emerging influenza season as data became available, and the tool predicted the upcoming season to be severe. In addition, the Flucast tool was modified and adapted for southern and northern hemisphere countries with regular seasonal patterns, and these are presented in Multimedia Appendices 5 and 6.

**Table 3:** Comparison of the five models and their corresponding seasonal influenza predictions.

Year		Actual impact of season	Prediction of seasonal impact by scoring criteria				
			Model 1	Model 2	Model 3	Model 4	Model 5
**2007**		Severe					
	Severity index		60	69	56	60	75
	Season prediction		Severe	Severe	Moderate	Severe	Severe
	Correct prediction		Yes	Yes	No	Yes	Yes
**2008**		Moderate					
	Severity index		50	50	56	55	35
	Season prediction		Moderate	Moderate	Moderate	Moderate	Moderate
	Correct prediction		Yes	Yes	Yes	Yes	Yes
**2009**		Very severe (pandemic)					
	Severity index		100	100	100	100	100
	Season prediction		Pandemic	Pandemic	Pandemic	Pandemic	Pandemic
	Correct prediction		Yes	Yes	Yes	Yes	Yes
**2010**		Mild					
	Severity index		25	25	25	30	30
	Season prediction		Mild	Mild	Mild	Moderate	Moderate
	Correct prediction		Yes	Yes	Yes	No	No
**2011**		Moderate					
	Severity index		35	38	31	40	45
	Season prediction		Moderate	Moderate	Moderate	Moderate	Moderate
	Correct prediction		Yes	Yes	Yes	Yes	Yes
**2012**		Severe					
	Severity index		65	63	63	65	75
	Season prediction		Severe	Severe	Severe	Severe	Severe
	Correct prediction		Yes	Yes	Yes	Yes	Yes
**2013**		Moderate					
	Severity index		35	38	31	40	35
	Season prediction		Moderate	Moderate	Moderate	Moderate	Moderate
	Correct prediction		Yes	Yes	Yes	Yes	Yes
**2014**		Moderate					
	Severity index		45	50	38	50	45
	Season prediction		Moderate	Moderate	Moderate	Moderate	Moderate
	Correct prediction		Yes	Yes	Yes	Yes	Yes
**2015**		Moderate					
	Severity index		55	63	44	60	65
	Season prediction		Moderate	Severe	Moderate	Severe	Severe
	Correct prediction		Yes	No	Yes	No	No
**2016**		Moderate					
	Severity index		35	44	25	40	35
	Season prediction		Moderate	Moderate	Mild	Moderate	Moderate
	Correct prediction		Yes	Yes	No	Yes	Yes
**2017**		Severe					
	Severity index		65	75	56	65	80
	Season prediction		Severe	Severe	Moderate	Severe	Severe
	Correct prediction		Yes	Yes	No	Yes	Yes
Predicted accuracy of past influenza seasons			11/11	10/11	8/11	9/11	9/11

## Discussion

The Flucast tool provides early prediction of seasonal severity of influenza using real-time data. In our prediction tool, we found that five parameters were optimal and that using more or fewer parameters reduced the predictive ability of the Flucast tool. The value of Flucast is in allowing real-time prediction of seasonal severity of an upcoming season with a simple tool to inform surge capacity planning. Also, the development of the tool using 11 years of historical influenza data and validation against the 2018 influenza season prospectively adds strength to the predictive value of the tool.

Routinely collected data from influenza surveillance schemes are regularly used by public health sectors to monitor epidemiological trends and to inform the burden of influenza-related illnesses for the planning of public health intervention programs in many countries. These may differ from country to country depending on health care resources, policy, and regulations adopted within the local context. Similar to Australian Influenza Surveillance Reports, the CDC in the United States, the European Centre for Disease Control and Prevention, and WHO regularly publish updated influenza surveillance data to inform current trends of influenza seasonal activity, circulating viral strains, disease impact on the health care system, and information regarding available vaccines and vaccination during the season and beyond. Although these data are descriptive and useful, they are not predictive. In this study, we have shown that it is possible to use the same data, combined with other parameters, to predict the seasonal severity of influenza. We understand that there are trade-offs between the use of sophisticated modeling techniques and a simple method in generating outputs for forecasting of influenza epidemics and outbreaks. Although studies have shown the potential benefits of using advanced modeling statistics in this area, most public health practitioners do not use such methods and rely on descriptive data [21]. There are many reasons for this, and some may be due to the lack of proper training or knowledge in modeling and uncertainty about modeling, which may hinder the efficient use of such tools [21]. There is also a need to engage health system stakeholders involved in operational response and to improve uptake of such tools for decision support.

There are some limitations to this study. First, the Flucast tool was developed using Australian data, and its application in other countries or settings was not evaluated. For example, in developing countries with limited surveillance capacity, all parameters may not be available. In some countries such as Thailand or Hong Kong, clear winter seasonality of influenza is not present, and there may be two peaks in the influenza seasons. These may have an impact on the predictivity of the Flucast tool. Secondly, there are variations in the influenza surveillance scheme and availability of country-specific data; thus not all parameters may be applicable in the Flucast tool. Thirdly, qualitative assessments of virulence and vaccine mismatch were made using reports from the surveillance system, and changes in these data during the season might influence the accuracy of output by the tool. Fourthly, inconsistency and variations in reporting and surveillance practices, as well as regional variation in influenza activity between jurisdictions, could limit the regional validity of the tool. Increased notifications over time may be due to wider availability and uptake of influenza tests, and they may not necessarily reflect the high incidence of infection, which can also vary subnationally. To overcome this problem in our study, we calibrated the input variables (that used laboratory-confirmed influenza notifications) based on the last 5 years’ average rather than using the prior years. In addition, we used the crude population death rate attributable to influenza reported per year to adjust for testing or reporting practices in the administrative data. Lastly, due to the unpredictable nature of influenza infections, predicting seasonal influenza activity can be complicated. It is driven by many factors, such as the viral strain in the season, vaccine mismatch with the circulating strain, vaccination coverage in the population, as well as environmental factors such as temperature and humidity. Therefore, care should be taken in interpreting the results. These should be continually revised using new data as it becomes available.

To conclude, Flucast is a simple tool and is intended to provide simple outputs for routine practice by public health officials in a real-time setting with minimal supervision. The tool can be used to plan for health care services and resources during the influenza season.

## Appendix

Multimedia Appendix 1 Model 2, with removal of a seasonal onset column from model 1.

Multimedia Appendix 2 Model 3, removal of an influenza-associated deaths column from model 1.

Multimedia Appendix 3 Model 4, using different scoring method for dominant circulating strain in model 1.

Multimedia Appendix 4 Model 5, using the number of influenza notifications in June instead of May in model 1.

Multimedia Appendix 5 The Flucast tool: parameters and scoring criteria for southern hemisphere countries.

Multimedia Appendix 6 The Flucast tool: parameters and scoring criteria for northern hemisphere countries.

## Abbreviations

CDC: Centers for Disease Control and Prevention
NNDSS: National Notifiable Diseases Surveillance System
WHO: World Health Organization
