# Supplementary material for: Flucast: A Real-Time Tool to Predict Severity of an Influenza Season
Source: JMIR Public Health Surveill. 2019 Jul 23;5(3):e11780. doi: 10.2196/11780 (PMC6683655; doi:10.2196/11780)
Supplement: Multimedia Appendix 1 [file publichealth_v5i3e11780_app1.docx]

Table 1. Model 2, with removal of a seasonal onset column from model 1.^a^

| Year | Actual impact of season | Parameters ^b^ | | | | Total scores (max=16) | Severity index,^c^ % |
| --- | --- | --- | --- | --- | --- | --- | --- |
|  |  | Relative magnitude of influenza activity (score) | Dominant strain in circulation (score) | Vaccine mismatch in the season (score) | Early season deaths (score) |  |  |
| 2007 | Severe | 1.3 (1) | A(H3N2) (3) | All strains (4) | 18^d^ (3) | 11 | **69** |
| 2008 | Moderate | 2.8 (4) | B (1) | 1 strain (2) | 3^d^ (1) | 8 | **50** |
| 2009 | Very severe (Pandemic) | 19.3 (4) | Novel / pandemic strain or A(H1N1)pdm09  (4) | All strains (4) | 61 (4) | 16 | **100** |
| 2010 | Mild | 0.3 (0) | A(H1N1)pdm09 (2) | None (1) | 2 (1) | 4 | **25** |
| 2011 | Moderate | 1.4 (1) | A(H1N1)pdm09  (2) | None (1) | 10 (2) | 6 | **38** |
| 2012 | Severe | 1.4 (1) | A(H3N2) (3) | >1 but not all strains (3) | 23 (3) | 10 | **63** |
| 2013 | Moderate | 0.8 (0) | A(H1N1)pdm09 (2) | 1 strain (2) | 11 (2) | 6 | **38** |
| 2014 | Moderate | 1.2 (1) | A(H1N1)pdm09  (2) | 1 strain (2) | 22 (3) | 8 | **50** |
| 2015 | Moderate | 2.9 (4) | B (1) | None (1) | 46 (4) | 10 | **63** |
| 2016 | Moderate | 1.5 (1) | A(H1N1)pdm09  (2) | None (1) | 17 (3) | 7 | **44** |
| 2017 | Severe | 2.1 (3) | A(H3N2) (3) | 1 strain (2) | 43 (4) | 12 | **75** |

^a^ Source: Australian Influenza Surveillance Reports [1] and NNDSS [2].

^b^ Timing of seasonal onset: ratio of laboratory-confirmed influenza notifications in May/January to April average [2]; relative magnitude of influenza activity: ratio of laboratory-confirmed influenza notifications in May compared to last 5 years’ average [2]; dominant strain: dominant strain in circulation [1]; vaccine mismatch in season: vaccine mismatch with dominant strain(s) [1]; early season deaths: rate per 100,000 population of notified influenza-associated deaths at the end of July in the season [1].

^c^ Severity index=total score/maximum score.

^d^ Influenza-associated deaths in 2007 and 2008 were estimated by calculating the proportion (total number of notifications at the end of July/total notifications in the year) multiplied by total deaths reported by the Australian Bureau of Statistics for 2007 and 2008, accordingly.

References

1. DoH: **Australian Government, Department of Health. Australian Influenza Surveillance Report and Activity Updates**. **Available at:** http://www.health.gov.au/flureport. Accessed 02 February, 2017. (Archived by WebCite^®^ at http://www.webcitation.org/71LeINYk2)

2. DoH: **Australian Government, Department of Health-National Notifiable Diseases Surveillance System Available at:** http://www9.health.gov.au/cda/source/cda-index.cfm. Accessed 03 July, 2017. (Archived by WebCite^®^ at http://www.webcitation.org/71LS3VfRM)
