# Supplementary material for: Flucast: A Real-Time Tool to Predict Severity of an Influenza Season
Source: JMIR Public Health Surveill. 2019 Jul 23;5(3):e11780. doi: 10.2196/11780 (PMC6683655; doi:10.2196/11780)
Supplement: Multimedia Appendix 5 [file publichealth_v5i3e11780_app5.docx]

Table 5. The Flucast tool: parameters and scoring criteria for southern hemisphere countries (assuming influenza season falls between May and October).

| Parameter and criteria | | | Scores |
| --- | --- | --- | --- |
| 1. Timing of seasonal onset | | |  |
|  | Ratio of laboratory-confirmed influenza notifications in month 1 (May) to preceding four months’ average for a given year, if: | |  |
|  |  | ≤1 | 0 |
|  |  | >1 to 1.5 | 1 |
|  |  | >1.5 to 2 | 2 |
|  |  | >2 to 2.5 | 3 |
|  |  | >2.5 | 4 |
| 2. Relative magnitude of influenza activity | | |  |
|  | Ratio of laboratory-confirmed influenza notifications in month 1 (May) for a given year compared with last 5 years’ average for the same period, if: | |  |
|  |  | ≤1 | 0 |
|  |  | >1 to 1.5 | 1 |
|  |  | >1.5 to 2 | 2 |
|  |  | >2 to 2.5 | 3 |
|  |  | >2.5 | 4 |
| 3. Dominant strain in circulation | | |  |
|  | Viral strain comprising ≥50% of circulating strains or the highest proportion circulating in the season | |  |
|  |  | B or A(H1N1) | 1 |
|  |  | A(H1N1)pdm09 | 2 |
|  |  | A(H3N2) | 3 |
|  |  | Novel strain | 4 |
| 4. Vaccine mismatch in the season | | |  |
|  | Documented vaccine mismatch with the dominant strain in the season | |  |
|  |  | No mismatch | 1 |
|  |  | Mismatch in 1 strain only | 2 |
|  |  | Mismatch in >1 but not all strains | 3 |
|  |  | Mismatch in all strains | 4 |
| 5. Early season deaths | | |  |
|  | Rate of notified influenza-associated deaths per 100,000 population at the end of July (month 3 or three months after the season starts) in the current season | |  |
|  |  | ≤0.01 | 1 |
|  |  | >0.01 to 0.05 | 2 |
|  |  | >0.05 to 0.1 | 3 |
|  |  | >0.1 | 4 |
